# Facilitation of Relational Learning in Schizophrenia 

**DOI:** 10.3390/bs3020206

**Published:** 2013-04-12

**Authors:** Elena A. Spieker, Jacqueline A. Griego, Robert S. Astur, Henry H. Holcomb, Laura M. Rowland

**Affiliations:** 1 Department of Psychiatry, Maryland Psychiatric Research Center, University of Maryland School of Medicine, P.O. Box 21247, Baltimore, MD 21228, USA; E-Mails: espieker@mprc.umaryland.edu (E.A.S.); jackiegriego@gmail.com (J.A.G.); hholcomb@mprc.umaryland.edu (H.H.H.); 2 Department of Psychology, University of Connecticut Waterbury, 99 East Main Street Waterbury, CT 06702, USA; E-Mail: Robert.Astur@uconn.edu; 3 Department of Psychiatry, Johns Hopkins University, 600 North Wolfe Street, Baltimore, MD 21287, USA; 4 Russell H. Morgan Department of Radiology and Radiological Science, Johns Hopkins University, 600 North Wolfe Street, Baltimore, MD 21287, USA

**Keywords:** schizophrenia, relational learning, training, transverse patterning, hippocampus, medial temporal lobe, memory

## Abstract

Abnormal hippocampal function likely contributes to relational learning deficits observed in schizophrenia. It is unknown whether these deficits can be attenuated with a training intervention. The purpose of this project was to determine if training could facilitate relational learning of the transverse patterning task in schizophrenia. Healthy and schizophrenia subjects completed a version of transverse patterning that incorporated training. The majority of subjects with schizophrenia successfully learned transverse patterning when provided with training. A subgroup (approximately 25%) of schizophrenia subjects showed no tendency to learn with training. These results were replicated in a second study with a separate cohort and different stimuli. This study illustrates that relational learning of the transverse patterning can be facilitated in schizophrenia with training.

## 1. Introduction

Hippocampal abnormalities figure prominently in the pathophysiology of schizophrenia [1]. These abnormalities presumably contribute to learning and memory deficits commonly associated with this disorder. Relational learning putatively relies on intact hippocampal function [2,3] and may be particularly vulnerable in schizophrenia. Studies of schizophrenia that revealed performance impairments in transitive inference [4,5] and the virtual Morris water task [6], assessments of relational learning, support this prediction. 

The transverse patterning (TP) problem provides a way to test relational memory [7]. It is similar to the childhood game “rock, paper, scissors”, and requires the subject to learn the relationship among three items. TP performance is dependent upon intact hippocampal function in a variety of species *i.e.*, rodents [8], primates [9,10], and humans [11,12]. Relevant to pathophysiology of schizophrenia, neonatal hippocampal lesions disrupt both TP learning and normal social behavior in adult monkeys [13]. Human studies of TP have revealed hippocampal BOLD signal changes with fMRI [14,15,16]. Hippocampal source activity with magnetoencephalography [17], and proton magnetic resonance spectroscopy neurochemical measures [18] also vary with TP performance. 

The purpose of this project was to determine if training could facilitate relational learning in subjects with schizophrenia. Healthy subjects learn the TP problem without training, when the three pairs are presented at once as in a standard task version [19]. Pilot work from our lab suggests that subjects with schizophrenia cannot learn the TP problem when provided with a standard version [20]. Therefore we instituted a stepwise training approach similar to one used in rodent, primate, and human studies [8,9,18,19].

Two studies were conducted with different subject cohorts. The purpose of the second study was to determine if the results were reproducible and if relational learning could be further facilitated when the stimuli were simplified and meaningful. One study using the TP task suggests performance is facilitated when “meaningful” stimuli are used [21]. The first study used abstract figures and the second study used common shapes as stimuli. The second study was conducted as part of a neuroimaging study and the neuroimaging data are presented elsewhere [16]. 

## 2. Methods

The inclusion/exclusion criteria for subjects with schizophrenia were: (1) diagnosis of schizophrenia as determined with the Structured Clinical Interview for DSM-IV, Patient Version (SCID-P) [22]; (2) no current or past neurological condition; (3) no DSM-IV substance abuse in the last six months; (4) clinically stable as determined by their treatment psychiatrist; and (5) same type and dose antipsychotic for at least three months. The inclusion/exclusion criteria for healthy volunteers were: (1) no past or present psychiatric disorder as determined with the Structured Clinical Interview for DSM-IV, Non-Patient Version (SCID-NP) [23]; (2) no first-degree relatives with a diagnosis of a psychotic disorder; (3) no current or past neurological condition. Subjects with schizophrenia were evaluated for their ability to provide informed consent before signing consent documents. All subjects gave written informed consent prior to participation in the study. This project was approved by the University of Maryland Internal Review Committee. 

### 2.1. Task

The task procedures have been described in animal and human studies [8,18,19] where two conditions, TP and simple discrimination (SD), were administered in a stepwise fashion. For the relational learning TP condition, subjects learned the relationship between three pairs of overlapping, ambiguous stimuli (e.g., A > B, B > C, C > A); and for the nonrelational learning SD condition subjects learned the simple discrimination of three pairs of nonoverlapping stimuli (e.g., D > E, F > G, H > I).

**Figure 1:**
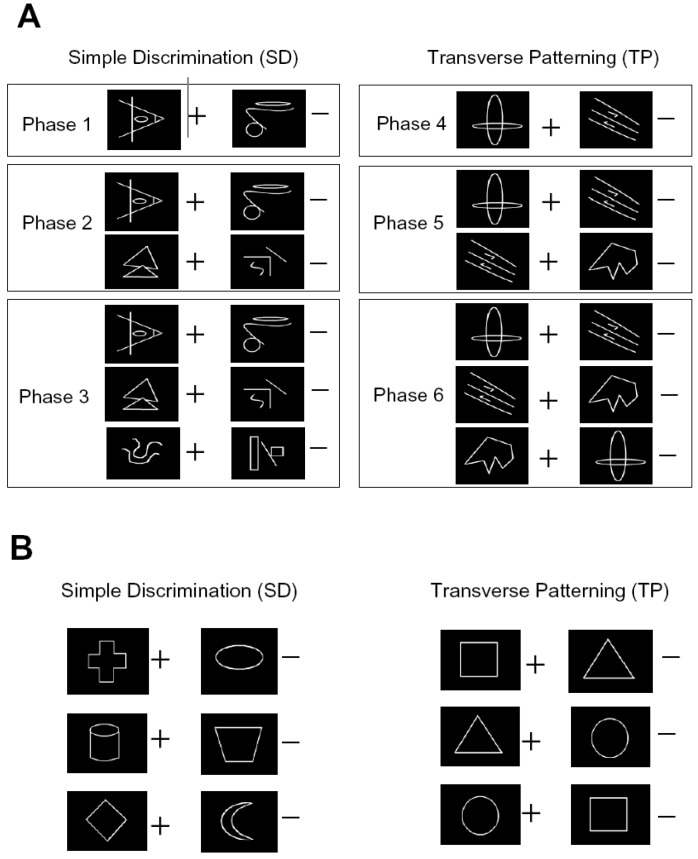
Illustration of the task. For a trial, one pair of stimuli illustrated above is presented. The subject’s goal is to learn the correct item in each pair. A “+” indicates the correct choice and a “−” indicates an incorrect choice. The transverse patterning (TP) condition requires relational learning, whereby an item is correct or incorrect depending on the item it is paired with. The simple discrimination (SD) condition does not require relational learning. The task comprised six phases. Task procedures were similar for Studies 1 and 2 except for the stimuli. (**a**) The abstract stimuli for Study 1 are illustrated in A. (**b**) The “shape” stimuli for Study 2 are illustrated in B.

Subjects completed six phases (see Figure 1 for illustration). Phases 1–3 pertained to the SD condition and Phases 4–6 pertained to the TP condition. Trials were presented in blocks that contained 16 trials ***per pair***. Advancement to the next phase required the subject to score 13 out of 16 correct responses ***per pair*** within a block. This criterion was important to ensure subjects were learning the entire problem and not a subset of it. A maximum of 10 blocks per phase was allowed. For Phases 1 and 4 which included one pair, the maximum number of trials was 160; for Phases 2 and 5 which included two pairs the maximum number of trials was 320; and Phases 3 and 6 which included three pairs the maximum number of trials was 480. The last Phase 6 of TP is considered hippocampal-dependent. The computerized task was created with EPRIME software (Psychology Software Tools, Inc.). 

Task procedures were identical for the two studies except for the stimuli. The task stimuli for study 1 were abstract figures and the task stimuli for study 2 were common shapes (see Figure 1).

### 2.2. Data Analyses

Trials to criterion were analyzed with 2(group) × 3(phase) ANOVAs, with repeated measures on phase, separately for the SD and TP conditions. Statistically significant interaction effects were followed up with post-hoc tests when appropriate. The relationships between number of trials to criterion and psychiatric measures were computed with Pearson’s product moment correlations. 

To examine if type of stimuli impacted performance, trials to criterion were analyzed with 2(group) × 2(study; abstract, shape) × 3(phase) ANOVAs, with repeated measures on phase, separately for the SD and TP conditions. All statistical tests were two-tailed. Analyses were conducted with the Statistical Package for Social Sciences (SPSS) version 12.0 [24].

## 3. Results

### 3.1. Study 1

Twenty subjects with a DSM-IV-TR diagnosis of schizophrenia (10 females; mean age 43 years) and 20 healthy control subjects (9 females, mean age 42 years) participated in this study. Subjects with schizophrenia were clinically stable (Brief Psychiatric Rating Scale [BPRS; 25]) total mean = 34.2 SD = 6.8) and all but three were treated with second-generation antipsychotic medication. The average level of function as determined by the Level of Function Scale (LOFS) was 23 (SD = 6.4). There was no significant difference in age, gender, handedness, or level of education between groups (all *p*’s > 0.05).

**Figure 2:**
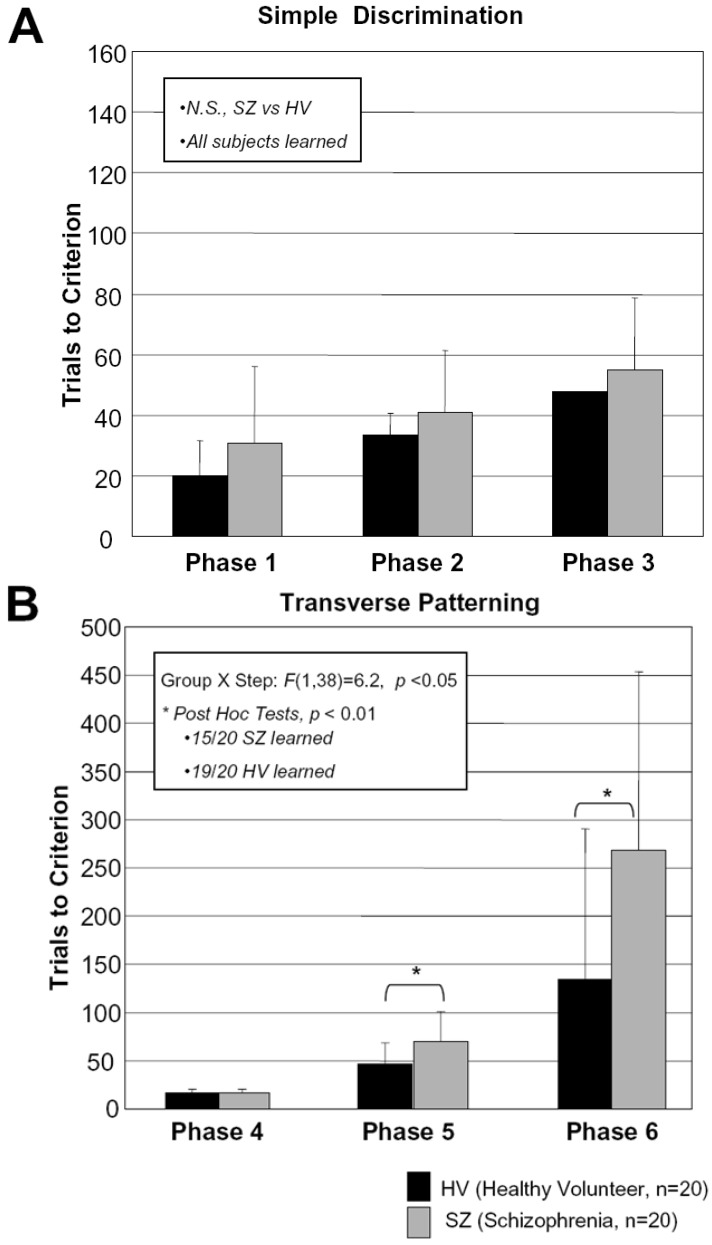
Means (SD) for number of trials to criterion for Study 1. (**a**) Trials to criterion for the SD condition that does not require relational learning. (**b**) Trials to criterion for the TP condition that is hippocampal-dependent and requires relational learning.

Means and standard deviations for TP and SD performance are presented in Figure 2. All subjects reached criteria for all phases of the nonrelational learning control condition (SD). There were no statistically significant differences between groups for the individual SD Phases 1–3. 

All subjects reached criteria for Phases 4 and 5 of the relational learning TP condition. There was a significant group X phase interaction (*F* = 6.2, *p* < 0.05, *df* = 1, 38). Post-hoc tests revealed that the groups performed similarly on Phase 4 but subjects with schizophrenia required more trials to reach criterion on Phases 5 (*t* = 2.86, *p* = 0.007, *df* = 1, 38) and 6 (*t* = 2.48, *p* = 0.018, *df* = 1, 38). It is important to note that even though subjects with schizophrenia as a group required more trials to reach criteria compared to controls on Phase 5, all subjects with schizophrenia successfully completed this phase. 

When the groups were divided into those who learned (*i.e*., reached criteria on Phase 6 (the hippocampal-dependent condition) *versus* those who did not learn, the majority of subjects with schizophrenia (75%, 15 out of 20) were able to learn the TP problem. Ninety-five percent (19 out of 20) of the control subjects reached criteria for TP. When “learners” only were examined, there were no statistically significant differences between control and schizophrenia “learners” on number of trials to reach criterion for TP Phase 6 but the magnitude of difference was moderate (Cohen’s *d* = 0.42).

To determine whether the “nonlearners” in the schizophrenia group showed a tendency toward any level of learning, the accuracy levels were determined for the first and last blocks in Phase 6. The nonlearners showed no tendency to learn. The mean (SD) accuracy for the first and last blocks was 49% (5) and 49.5% (3.6), respectively. To determine if nonlearners showed a tendency to learn the last pair (*i.e.*, pair number 3) in Phase 6, the accuracy levels were determined for the individual TP pairs. The nonlearners showed no tendency to learn the last pair and showed a performance decrease for pairs 1 and 2. The mean (SD) accuracy for TP pairs 1, 2, and 3 was 51.2% (14.3), 51.1% (14.4), 51.6% (15.9), respectively.

There were no significant relationships between the number of trials to reach criterion for TP (Phase 6) or SD (Phase 3) and psychiatric symptoms (BPRS total, positive and negative scales or LOFS). 

### 3.2. Study 2

Seventeen subjects with a DSM-IV-TR diagnosis of schizophrenia (8 females; mean age 42 years) and 17 healthy control subjects (8 females, mean age 41 years) participated in this study. Subjects with schizophrenia were clinically stable (BPRS total mean = 31 SD = 6.3) and all but four were treated with second-generation antipsychotics. The average level of function as determined by the LOFS was 24.2 (SD = 5.8). Subjects with schizophrenia had fewer years of education (*t* = 2.59, *p* < 0.05, *df* = 32). There were no significant differences in age or gender between groups (all *p*’s > 0.05). 

**Figure 3:**
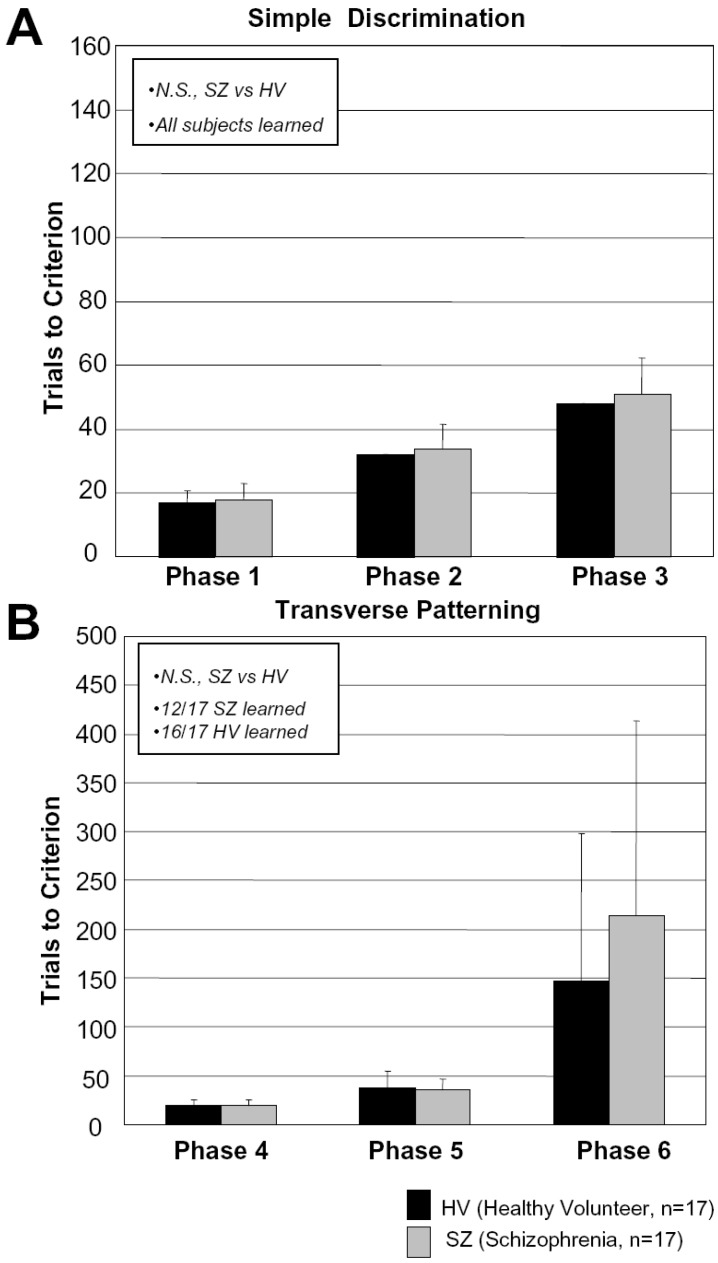
Means (SD) for number of trials to criterion for Study 2. (**a**) Trials to criterion for the SD condition. (**b**) Trials to criterion for the TP condition.

Means and standard deviations for TP and SD performance are presented in Figure 3. All subjects reached criteria for all phases of the nonrelational learning control condition (SD) and Phases 4 and 5 of the relational learning TP condition. There were no statistically significant or marginal differences between groups for any of the individual phases (all *p*’s > 0.27). 

Similar to study 1, the majority of subjects with schizophrenia (71%, 12 out of 17) were able to learn the TP problem (*i.e*., reached criteria on Phase 6 the hippocampal-dependent condition). Ninety-four percent (16 out of 17) of the control subjects reached criteria for TP.

To determine whether the “nonlearners” in the schizophrenia group showed a tendency toward any level of learning, the accuracy levels were determined for the first and last blocks in Phase 6. The nonlearners showed no improvement from the first to the last block. The mean (SD) accuracy for the first and last blocks was 60.8% (6.7) and 57.1% (10.8), respectively. To determine if nonlearners showed a tendency to learn the last pair (*i.e*., pair number 3) in Phase 6, the accuracy levels were determined for the individual TP pairs. The nonlearners showed no tendency to learn the last pair and showed a slight decrement in accuracy for pair 2. The mean (SD) accuracy for TP pairs 1, 2, and 3 was 85.3% (15.6), 56.1% (18.4), 27.1% (15.8), respectively. 

Consistent with Study 1, there were no significant relationships between the number of trials to reach criterion for TP (Phase 6) or SD (Phase 3) and psychiatric symptoms (BPRS total, positive and negative scales or LOFS). 

### 3.3. Study 1 and Study 2 Comparison

For the SD condition, there were no significant interactions but there was a significant main effect of study (*F* = 5.22, *p* = 0.025, *df* = 1, 70) indicating a greater number of trials to criterion for study 1 compared to study 2 averaged across group and phase. There was also a significant main effect of group (*F* = 5.93, *p* = 0.017, *df* = 1, 70) indicating a greater number of trials to criterion for schizophrenia compared to healthy controls averaged across study and phase.

For the TP condition, there were no significant interactions or main effect with study included as a between subjects factor (all *p*’s > 0.4). Therefore, the type of stimuli did not significantly impact TP performance. There was, however, a group X phase interaction (*F* = 6.34, *p* = 0.014, *df* = 1, 70). Post-hoc follow-up tests revealed greater number of trials to criterion for the hippocampal-dependent Phase 6 in the schizophrenia compared to the control group (*t* = 2.59, *p* = 0.012, *df* = 1, 72).

## 4. Discussion

Relational learning deficits have been frequently reported in schizophrenia [4,5,6,26,27] but the impact of training is unknown. The purpose of this project was to determine if training could facilitate relational learning of the transverse patterning task in schizophrenia. Results from two separate cohorts showed that the majority of subjects with schizophrenia successfully achieved relational learning proficiency when provided with training. Moreover there was a subgroup of subjects with schizophrenia whose performance remained impaired despite the training intervention. 

Extensive research suggests that stepwise TP learning requires intact hippocampal function [8,9,10,11,12,13]. The subgroup of subjects with schizophrenia that failed to learn the TP problem may have a severely dysfunctional hippocampal system. Supporting this supposition, two fMRI studies reported differences in hippocampal activation between schizophrenia and control groups during transitive inference [4] and transverse patterning [16] tasks. However, high and low performers with schizophrenia were not compared in these studies. Results of a pilot magnetoencephalography (MEG) study showed enhanced bilateral hippocampal source activation during successful (89% accuracy) TP learning in nine subjects with schizophrenia compared to controls [28]. Future neuroimaging studies may help determine whether performance differences between learner and nonlearner subjects with schizophrenia can be explained by brain structural, chemical or functional differences.

Another plausible explanation is that those subjects with schizophrenia with successful relational learning do so through alternative, extra-hippocampal brain networks. Literature on neuroplasticity, rehabilitation, and aging [29,30] supports the concept that if a brain region of the network is faulty, other brain regions compensate for its diminished function. With respect to RL, one study reported successful RL in an amnesic patient [31]. This patient was able to successfully perform a RL task by relying on semantic knowledge strategy despite having extensive bilateral MTL damage. Hence, it is plausible that RL can be accomplished through extra-MTL brain regions. It is reasonable to suppose there may be a subgroup of subjects with schizophrenia that perform normally on relational learning paradigms if provided with a training intervention. 

In both studies there were subgroups of subjects with schizophrenia that did not learn the TP problem in spite of ample trial opportunities. These findings are consistent with the concept that subjects with schizophrenia fall into subgroups of good and poor learners [32,33,34,35]. Given that nonlearners were as proficient as learners on the SD task, it is unlikely that poor TP performance is due to a generalized cognitive deficit. Moreover, the nonlearners did not differ in demographic, medication status, or psychiatric symptom measures from the learners with schizophrenia. Overall these findings lend further support to the heterogeneity of schizophrenia.

Contrary to expectation the simplified, recognizable stimuli employed in Study 2 did **not** further facilitate learning performance in subjects with schizophrenia who successfully learned the TP problem. It is possible that as long as each stimulus is unique and easily distinguished the nature of the stimuli, abstract or concrete, has little impact. Anecdotal evidence from post-task debriefing supports this notion since most subjects, in both diagnostic groups reported assigning names to the abstract stimuli as part of their cognitive strategy. However, it is possible that simplified, recognizable stimuli did facilitate learning performance in the nonlearner subjects with schizophrenia despite them not learning the full TP problem. 

There are some study considerations. First, antipsychotic medications may affect learning. However, the majority of subjects with schizophrenia were taking second-generation antipsychotic medications. Three subjects with schizophrenia were taking first-generation antipsychotic medications in Study 1 and four in Study 2. All subjects with schizophrenia had been treated with the same dose of antipsychotic medication for at least three months immediately prior to the study. The seven subjects taking first generation antipsychotics did not differ on psychiatric symptom ratings or learning performance when compared to the subjects taking second-generation antipsychotics. Second, one could argue that the subjects with schizophrenia who did not successfully learn the TP problem simply perform worse on tasks that are more difficult. This cannot be fully ruled out. However, these subjects did not differ from the successful patient learners on the Phase 3 of the control task that was matched for the number of stimulus pairs as Phase 6 of the TP problem, nor did they differ on Phase 5 of the TP problem. 

## 5. Conclusions

By focusing on a training program that facilitates learning success this study contributes to an emerging view of learning in schizophrenia. Subjects with schizophrenia may be able to reach remarkably normal cognitive skills when engaged in systematic, reinforced training programs [36,37] in spite of their having aberrant neural strategies [16,38]. This concept may be important since learning potential is related to rehabilitation outcome in schizophrenia [39].
